# Infant liver biochemistry is different than current laboratory accepted norms

**DOI:** 10.1007/s00431-023-05248-x

**Published:** 2023-10-09

**Authors:** Kaija-Leena Kolho, Tapio Lahtiharju, Laura Merras-Salmio, Mikko P. Pakarinen, Mikael Knip

**Affiliations:** 1 Department of Pediatric Gastroenterology, Children’s Hospital, University of Helsinki, P.O. Box 281, FI-00029 Helsinki, Finland; 2 Department of Clinical Laboratory, HUSLAB, Children’s Hospital, University of Helsinki, Helsinki, Finland; 3 Department of Pediatric Surgery, Children’s Hospital, University of Helsinki, Helsinki, Finland

**Keywords:** Alkaline phosphatase, ALT, AST, Babies, Bilirubin, Children

## Abstract

The purpose is to study liver biochemistry in a well-defined cohort of term infants. The methods include healthy term infants (*n* = 619) provided blood samples at 3 and 6 months of age when participating to the DIABIMMUNE study. The infants were followed up at clinical study visits 3, 6, 12, 18, 24, and 36 months the participation rate being 88.6% at the end of follow-up, while none disclosed any signs of a liver disease. The serum levels of serum alanine aminotransferase (ALT), aspartate aminotransferase (AST), alkaline phosphatase (ALP), gamma-glutamyl transferase (GGT), total bilirubin (BIL), and conjugated bilirubin (BIL-conj) were determined using Siemens Atellica CH 930 analyzers. The results are at 3 months of age, the upper 90% CI for ALT, AST, ALP, GGT, BIL, and BIL-conj were higher than the current upper reference limits in our accredited hospital laboratory. At 6 months, the upper 90% CIs for ALT had declined but was still higher than the cut-offs for a raised value. The upper 90% CI for AST remained as high as at 3 months, whereas ALP, BIL-conj, and GGT had decreased close to the current cut-offs. The type of feeding was associated with the levels of liver biochemistry. Exclusively or partially breastfed infants showed higher ALT, AST, BIL, and BIL-conj values at 3 months than formula-fed. Breastfed infants had higher AST, Bil, and Bil-conj values also at 6 months.

*  Conclusion*: We encourage setting appropriate reference ranges for liver biochemistry for the first year of life and to note the type of feeding.

**Table:** 

**What is Known:** *• Healthy infants may show higher values of liver biochemistry during their first year of life than in later life.* *• It has been speculated that type of feeding may play a role in liver biochemistry levels among infants.*	
**What is New:** *• In a cohort of healthy infants, several analytes of liver biochemistry were higher than the currently used upper reference limits at 3 and 6 months of age, and exclusively or partially breastfed infants showed higher values than formula-fed.* *• The findings address the importance of setting appropriate reference ranges for liver biochemistry for the first year of life.*

**What is Known:**

*• Healthy infants may show higher values of liver biochemistry during their first year of life than in later life.*

*• It has been speculated that type of feeding may play a role in liver biochemistry levels among infants.*

**What is New:**

*• In a cohort of healthy infants, several analytes of liver biochemistry were higher than the currently used upper reference limits at 3 and 6 months of age, and exclusively or partially breastfed infants showed higher values than formula-fed.*

*• The findings address the importance of setting appropriate reference ranges for liver biochemistry for the first year of life.*

## Introduction

We observed that in full-term unjaundiced infants with uneventful growth and development presenting with mildly elevated levels of serum liver biochemistry, any underlying hepatobiliary disorders are hardly ever disclosed. This prompted us to take a closer look at liver biochemistry levels in a well-defined cohort of healthy term infants. Included babies were initially recruited to the DIABIMMUNE study assessing risk for development of type 1 diabetes [1]. We utilized blood samples collected at the age of 3 and 6 months. We hypothesized that the current liver biochemistry reference values are not appropriate for infants within the first months of life, while more accurate cut-offs for raised values are needed. Notably, breastfeeding may impact the live biochemistry of infants.

## Methods

The blood samples were obtained from babies initially recruited to the DIABIMMUNE study assessing risk for development of type 1 diabetes, as described in detail [1]. The parents reported using a structured questionnaire the type of feeding as exclusive or partial breastfeeding or as formula feeding and use of supplementary foods and elimination diets, listed medications, and hospitalizations. We utilized blood samples collected at the age of 3 and 6 months at the time of study visits including clinical examination. Duration of exclusive and partial breastfeeding was reported in months (with two decimals). The samples were collected between Sept 2008 and March 2010, stored in − 70 °C, and analyzed between Aug and Dec 2021. All the samples were analyzed for serum alanine aminotransferase (ALT), aspartate aminotransferase (AST), alkaline phosphatase (ALP), gamma-glutamyl transferase (GGT), total bilirubin (BIL), and conjugated bilirubin (BIL-conj) and albumin (ALB) in the accredited laboratory of Helsinki University Hospital using Siemens Atellica CH 930 analyzers, to ALT and AST assays we added pyridoxal-5-phosphate (P5P). C-reactive protein (CRP) was also measured to exclude acute infections. The stability of the measured values during storage is considered good.

The infants were followed up for 3 years (including study visits and clinical examination at 3, 6, 12, 18, 24, and 36 months the participation rate being 88.6% at the end of follow-up), and none presented with any hepatobiliary disease [1]. Of the mothers, 69 (11%) had a diagnosis of a chronic disease (asthma *n* = 35, thyroid disease *n* = 21, celiac disease *n* = 3, type 1 diabetes *n* = 6, and type 2 diabetes *n* = 4) and 6 (0.96%) presented with gestational diabetes.

### Statistical analyses

We tested the need for partitioning between sexes and nutritional status with Harris and Boyd method (see the guideline of Clinical and Laboratory Standards Institute (CLSI) [2]). To define reference limits and their 90% confidence intervals (90% CI), we followed the CLSI [2]. We used the non-parametric method for groups of over 120 participants and the robust method of Horn and Pesce for others. Except for the conjugated bilirubin (BIL-conj) of non-breastfed infants, where most results were below measuring range not allowing estimation of CIs, we defined the percentiles of 2.5% and 97.5% from the results. We removed outliers from the analysis using the modified Dixon method as explained [2].

## Results

The study population included 619 healthy infants: 322 were 3-month-old, and 297 were 6-month-old at sampling; 252 were sampled at both time points. We excluded 42 infants with CRP > 4 mg/L. The laboratory tests are shown in Table 1. At 3 months of age, the upper 90% CI for serum ALT, AST, ALP, GGT, BIL, and BIL-conj were higher than the current upper reference limits in our accredited hospital laboratory. At 6 months, the upper 90% CIs for ALT and ALP had declined but were still higher than the current cut-offs for a raised value. The upper 90% CI for AST remained as high as at 3 months, but ALP and BIL-conj had decreased close to the current cut-offs (Table 1). The only biochemical analyte showing statistically lower upper limit was ALB at 3 months of age; at 6 months, the upper limit was still numerically lower without statistical significance.

**Table 1:** Liver biochemistry in serum at the age of 3 and 6 months. Lower limit (LL) and upper limit (UL) are the estimated reference range. Current LL and current UL are the current reference limits used in our accredited laboratory for the specified age. LL and UL that do not have current LL or UL, respectively, within the 90% confidence interval are in bold. The *p*-value of nutrition is the significance of difference between medians of breastfed and not breastfed infants, and the statistically significant differences are in bold

**Biomarker**	**Age months**	**All infants**						**Breastfed infants**				**Infants not breastfed**				***p*-value of nutrition**
		***n***	**Median (IQR)**	**LL (90% CI)**	**Current LL**	**UL (90% CI)**	**Current UL**	***n***	**Median (IQR)**	**LL (90% CI)**	**UL (90% CI)**	***n***	**Median (IQR)**	**LL(90% CI)**	**UL (90% CI)**	
Alb (g/L)	3	296	41 (39–43)	37 (34–37)	37	**47** (46–48)	**51**	226	41 (39–43)	37 (35–37)	47 (46–48)	48	41 (40–43)	36 (36–37)	46 (45–47)	0.86
	6	274	43 (41–45)	37 (37–37)	37	49 (48–52)	51	174	43 (41–45)	37 (37–38)	49 (47–51)	99	43 (41–45)	37 (36–38)	49 (48–50)	0.79
ALT (U/L)	3	296	35 (22–49)	< 9 (< 9– < 9)	**-**	**96** (86–105)	**40**	226	36 (24–52)	< 9 (< 9– < 9)	97 (86–112)	50	30 (11–42)	< 9 (< 9– < 9)	72 (63–81)	**0.006**
	6	273	32 (23–42)	< 9 (< 9– < 9)	**-**	**75** (72–183)	**40**	175	33 (22–44)	< 9 (< 9– < 9)	88 (71–183)	97	31 (24–37)	< 9 (< 9– < 9)	69 (64–73)	0.28
AST (U/L)	3	293	51 (43–63)	31 (28–33)	**-**	**96** (92–102)	**50**	224	53 (43–65)	33 (30–36)	96 (92–105)	49	45 (38–51)	24 (20–28)	65 (60–70)	** < 0.001**
	6	272	52 (44–65)	34 (31–35)	**-**	**97** (89–155)	**50**	173	56 (48–67)	35 (31–39)	116 (89–155)	98	48 (42–55)	32(28–35)	80 (74–106)	** < 0.001**
ALP (U/L)	3	298	291 (234–342)	**138 (124–154)**	**115**	**485** (461–518)	**460**	228	294 (241–350)	153 (124–168)	509 (453–545)	50	288 (203–326)	86 (51–129)	458 (421–488)	0.068
	6	278	257 (209–309)	123 (106–128)	115	467 (430–538)	460	179	241 (192–298)	116 (106–127)	417 (404–465)	98	286 (232–328)	101 (74–131)	448 (418–477)	** < 0.001**
GGT (U/L)	3	296	25 (20–33)	11 (10–12)	**-**	**65** (61–85)	**50**	226	25 (20–33)	12 (10–13)	65 (58–93)	50	25 (21–32)	< 7 (< 7– < 7)	54 (46–62)	0.82
	6	274	18 (14–22)	9 (< 7–10)	**-**	**40** (37–45)	**50**	175	18 (15–23)	8 (< 7–11)	40 (33–45)	98	17 (14–20)	< 7 (< 7– < 7)	31 (28–34)	0.12
BIL (µmol/L)	3	298	7 (4–9)	< 3 (< 3– < 3)	**-**	**32** (25–35)	**20**	228	7 (4–10)	< 3 (< 3– < 3)	32 (25–41)	50	4 (< 3–7)	< 3 (< 3– < 3)	12 (10–14)	** < 0.001**
	6	278	5 (3–7)	< 3 (< 3– < 3)	-	15 (13–24)	20	179	5 (4–8)	< 3 (< 3– < 3)	16 (13–24)	98	4 (< 3–5)	< 3 (< 3– < 3)	10 (9–11)	** < 0.001**
BIL-conj (µmol/L)	3	293	2 (< 2–4)	< 2 (< 2– < 2)	**-**	**10** (9–12)	**5**	224	2 (< 2–4)	< 2 (< 2– < 2)	10 (9–14)	49	< 2 (< 2–3)	< 2*	5*	**0.002**
	6	271	< 2 (< 2–3)	< 2 (< 2– < 2)	-	6 (5–9)	5	173	< 2 (< 2–3)	< 2 (< 2– < 2)	7 (5–9)	98	< 2 (< 2–2)	< 2*	4*	**0.003**

For all and breastfed infants, the range was calculated with non-parametric method and for infants not breastfed was calculated with robust method or in *case of conjugated bilirubin (BIL-conj) with estimating directly the 2.5% and 97.5% percentiles (see ref 2). With all patients, there were one outlier in 6-month-old ALP, GGT, BIL, and BIL-conj. With breastfed patients, there were one outlier in 6-month-old ALP and GGT, and with patients without breastfeeding, there were two outliers in 3-month-old Alb and one in 6-month-old BIL. Current reference limits used in our accredited laboratory for the specified age are available at https://huslab.fi/ohjekirja/ (text in Finnish)

*Alb* albumin, *ALT* alanine aminotransferase, *AST* aspartate aminotransferase, *ALP* alkaline phosphatase, *GGT* gamma-glutamyl transferase, *BIL* total bilirubin, *BIL-conj* conjugated bilirubin, *IQR* inter-quartile range, *LL* lower limit, and *UL* upper limit

The type of feeding was associated with the levels of liver biochemistry, and exclusively or partially breastfed infants showed higher values than formula-fed for ALT, AST, BIL, and BIL-conj at 3 months and for AST, BIL, and BIL-conj at 6 months (Table 1). There was no statistically significant difference in the upper limits of GGT between breastfed and bottle-fed children (the 90% CIs overlap and a *p*-value of 0.82 from the Wilcoxon rank test).

## Discussion

Biological variation is the key phenomenon in interpretation of laboratory results and selecting accurate reference values [3]. Optimally, a raised value would indicate further investigations and an underlying disease with high accuracy, and likewise, a value within normal range would exclude the possibility of a given disease. In pediatrics, individual development of children further challenges the definitions of abnormality. Here, we showed that the current cut-offs for raised liver biochemistry values are too low during the first 6 months of life. Importantly, breastfeeding resulted in somewhat higher values of ALT, AST, BIL, and BIL-conj. We speculate that this is due to exposure of maternal hormones via breastmilk [4, 5].

Previously, AST and total BIL were shown to be higher in breastfed infants compared to formula-fed infants at 2 and 6 months of age. The study cohort was smaller than ours and included samples of 71 and 58 infants, respectively. In contrast to our study, there was no such difference in ALP levels, and ALT was not measured [6]. A larger study of 2000 infants reported higher ALT levels during the first year of life than in older children, the first-year values being comparable to our findings [7]. They did not measure AST, and type of feeding was not recorded [7]. More recently, pediatric reference intervals for biochemical markers including ALB, ALP, ALT, AST, GGT, and BIL were defined in a Canadian cohort of 600 samples from 0 to 18-year-olds [8]. They used Siemens Atellica analyzer, but it is not clear if P5P was used or not. In our study, the lower limit of ALB and upper limits of ALT, GGT, BIL, and BIL-conj were different. However, in the Canadian study, only ALP and GGT results (less than 60 samples) were stratified according to 6 months age limit. Moreover, there were no data related to breastfeeding or the health outcomes of the infants. At 1 year, the liver biochemistry did not show as high values as we observed [8]. Our study showed that the age partitions of 6 months or 1 year used by Bohn et al. [8] were not rigorous enough. Also, the status of breastfeeding had an impact on liver biochemistry at least up to 6 months of age.

In line with this, age- and gender-appropriate reference values were established for 15 biochemical analytes in apparently healthy Chinese children and adolescents using Olympus AU5400 analyzer. Regarding liver biochemistry, gender partitions were required for ALT, AST, GGT, and ALP [9]. Although their absolute values differed from ours, they observed as well that young infants had higher values of liver biochemistry [9]. Feeding data and whether they used P5P were not available. In a previous Chinese study of 60 infants studied at weeks 4 and 8, at a younger age than in our cohort, breastfed infants had higher levels of ALT, AST, GGT, and BIL as well [10]. The health of the infants was not followed up. Our follow-up extended up to 3 years of life, and there was no indication to further investigations on liver function in any of the children [1]. A recent review suggested to conclude follow-up of an asymptomatic patient with normal physical examination and normal values of liver function tests in the presence of two consecutive normal tests 1 month apart [11].

Correct identification of cholestasis is critical among neonates and infants. According to US guidelines, the cut-off for cholestasis evaluation is S-BIL-conj > 17 µmol/l that is clearly above the 90% CI of healthy infants we observed here, reassuring that with these cut-offs the risk for missing underlying hepatobiliary pathology is not increased [12].

As a limitation, silent vertical transmission of infectious agents, e.g., CMV or other viruses affecting liver, could not be excluded. However, such diseases are rare, and none of the mothers was with a diagnosis of hepatitis.

To conclude, healthy infants may display elevated liver biochemistry values at 3 and 6 months of age in comparison to later stages of life. Notably, breastfeeding influences the levels of ALT, AST, BIL, and BIL-conj resulting in higher values during the initial months of life. This observation holds significance as it can help to avoid unnecessary investigations and alleviate the burden on families. However, it is important to acknowledge the limitation that our study only had access to samples up to 6 months of age, and no further assessments of liver function were conducted. Nevertheless, we monitored the infants until 3 years of age (the age of last follow-up visit), and none exhibited any indications of liver disease. We advocate for the establishment of appropriate reference ranges for liver biochemistry during the first year of life.

## Data Availability

Data available on a reasonable request.

## Abbreviations

ALT: Serum alanine aminotransferase
AST: Aspartate aminotransferase
ALP: Alkaline phosphatase
BIL: Total bilirubin
BIL-conj: Conjugated bilirubin
GGT: Gamma-glutamyl transferase
